# Gut Microbiota Offers Universal Biomarkers across Ethnicity in Inflammatory Bowel Disease Diagnosis and Infliximab Response Prediction

**DOI:** 10.1128/mSystems.00188-17

**Published:** 2018-01-30

**Authors:** Youlian Zhou, Zhenjiang Zech Xu, Yan He, Yunsheng Yang, Le Liu, Qianyun Lin, Yuqiang Nie, Mingsong Li, Fachao Zhi, Side Liu, Amnon Amir, Antonio González, Anupriya Tripathi, Minhu Chen, Gary D. Wu, Rob Knight, Hongwei Zhou, Ye Chen

**Affiliations:** a Department of Gastroenterology, State Key Laboratory of Organ Failure Research, Guangdong Provincial Key Laboratory of Gastroenterology, Nanfang Hospital, Southern Medical University, Guangzhou, China; b Department of Environmental Health, School of Public Health and Tropical Medicine, State Key Laboratory of Organ Failure Research, Southern Medical University, Guangzhou, China; c Department of Gastroenterology, Guangzhou Digestive Disease Center, Guangzhou First People’s Hospital, Guangzhou Medical University, Guangzhou, China; d Department of Pediatrics, University of California, San Diego, La Jolla, California, USA; e Institute of Digestive Diseases, Chinese PLA General Hospital, Beijing, China; f Department of Gastroenterology, the First Affiliated Hospital, Sun Yat-Sen University, Guangzhou, China; g Perelman School of Medicine, University of Pennsylvania, Philadelphia, Pennsylvania, USA; h Department of Computer Science and Engineering, University of California, San Diego, La Jolla, California, USA; i Center for Microbiome Innovation, University of California, San Diego, La Jolla, California, USA; j State Key Laboratory of Organ Failure Research, Division of Laboratory Medicine, Zhujiang Hospital, Southern Medical University, Guangzhou, China; k School of Food and Technology, Nanchang University, Nanchang, China; l State Key Laboratory of Food Science and Technology, Nanchang University, Nanchang, China; University of Naples Federico II

**Keywords:** gut microbiota, infliximab treatment, disease activity, inflammatory bowel disease

## Abstract

Gut microbiota dysbiosis contributes to the onset and perpetuation of inflammatory bowel disease (IBD). Given that gut microbiotas vary across geography and ethnicity, it remains obscure whether any universal microbial signatures for IBD diagnosis and prognosis evaluation exist irrespective of populations. Here we profiled the fecal microbiota of a series of Chinese IBD patients and combined them with two Western IBD cohorts, PRISM and RISK, for meta-analyses. We found that the gut microbial alteration patterns in IBD are similar among Chinese and Westerners. Our prediction model based on gut microbiome for IBD diagnosis is robust across the cohorts, which showed 87.5% and 79.1% prediction accuracy in Crohn’s disease (CD) and ulcerative colitis (UC) patients, respectively. A relative increase in the levels of *Actinobacteria* and *Proteobacteria* (*Enterobacteriaceae*) and a relative decrease in the levels of *Firmicutes* (*Clostridiales*) were strongly correlated with IBD severity (*P* < 0.05). Additionally, restoration of gut microbiota diversity and a significant increase in *Clostridiales* relative abundance were found in patients responding to infliximab (IFX [Remicade]) treatment compared to those in relapse. Moreover, certain microbes, mainly *Clostridiales*, predicted the treatment effectiveness with 86.5% accuracy alone and 93.8% accuracy in combination with calprotectin levels and Crohn’s disease activity index (CDAI). Taking the results together, we conclude that gut microbiota can offer a set of universal biomarkers for diagnosis, disease activity evaluation, and infliximab treatment response prediction in IBD.

**IMPORTANCE** In the present report, we show that the human fecal microbiota contains promising and universal biomarkers for the noninvasive evaluation of inflammatory bowel disease severity and IFX treatment efficacy, emphasizing the potential ability to mine the gut microbiota as a modality to stratify IBD patients and apply personalized therapy for optimal outcomes.

## Abstract (precis)

In the present report, we show that the human fecal microbiota contains promising and universal biomarkers for the noninvasive evaluation of inflammatory bowel disease severity and IFX treatment efficacy, emphasizing the potential ability to mine the gut microbiota as a modality to stratify IBD patients and apply personalized therapy for optimal outcomes.

## INTRODUCTION

Inflammatory bowel diseases (IBD) are chronic relapsing inflammatory disorders that have been categorized into two main clinical phenotypes: ulcerative colitis (UC) and Crohn’s disease (CD). The global prevalence of IBD has been rising significantly, largely paralleling industrialization, with a concurrent increase in health care costs (1). Although their etiology and pathogenesis are not fully understood, genetic polymorphisms associated with IBD suggest an underlying role of aberrant immune responses to imbalanced gut microbiota as a key mechanism involved in disease pathogenesis (2, 3). Genome-wide association studies (GWAS) have identified more than 160 single nucleotide polymorphisms (SNPs) associated with IBD, many of which are involved in pathways that modulate the host response to microbial stimuli. The NOD2 gene, the first gene identified to have an association with IBD susceptibility, recognizes components of the bacterial cell membrane (4, 5). In patients with IBD, the diversity of the fecal microbiome is reduced compared to that of healthy controls (HC) (6). The major changes in the diversity of gut microbiota in the context of new-onset CD (before treatment is initiated) are correlated strongly with disease status (7–9). A broad pattern that differentiates IBD patients from healthy individuals has begun to emerge: reduced biodiversity, decreased abundance of several taxa in the *Firmicutes* phylum, and increased abundance of *Gammaproteobacteria* (10–14). Such alterations are consistent with the response of a complex microbial community to environmental stressors introduced by the host inflammatory response such as the production of alternative electron acceptors that promote nitrate respiration (15), as well as of oxygen radicals leading to oxidative stress (11). However, most of the aforementioned studies have been conducted in North American, European, and Japanese populations, whose genetics, ethnic backgrounds, environments, dietary habits, and lifestyles differ from those in China (16). It is well known that the gut microbiotas of human populations residing across different geographical locations are associated with significant differences in microbiota composition (17). However, it is currently unknown whether a disease state such as IBD would modify the composition of the gut microbiota in a consistent fashion independently of these geographical influences.

The natural history of CD is characterized by poorly predictable phases of quiescence and activity (18). In the absence of reliable predictors at the individual level, optimal medical strategies (e.g., top-up versus top-down management) remain debatable. The use of Infliximab (IFX [Remicade]), a human/mouse chimeric monoclonal antibody targeting tumor necrosis factor alpha (TNF-α) (19), is an effective treatment for patients with refractory moderate to severe CD. Nevertheless, some patients do not respond to IFX or else relapse after initial response. Our previous studies had suggested that the relapse rates were 10.53% in CD (20) and 25.0% in UC (21) at week 22 and were much higher after 30 weeks posttreatment. The inability to effectively predict the long-term efficacy of IFX treatment prevents adoption of a more targeted approach to the use of this class of biological agents with the intent of reducing both costs and the potential incidence of adverse events.

The choice of therapy is currently driven primarily by clinical predictive factors (22–24). Fecal calprotectin, a cytosolic protein of mucosal neutrophils that are extruded into the gastrointestinal (GI) tract when they undergo apoptosis (25), is useful to predict relapse in IBD patients (23, 26). Patients with high C-reactive protein (CRP) levels are more likely to achieve and maintain a response to biological therapy than those with low or normal CRP levels (27). Recently, the gut microbiota has gained attention as a reservoir of numerous microbial markers. It was found that reduced *Firmicutes* abundance is correlated with a shorter time to relapse after IFX withdrawal (28). The abundance of six clades of bacteria, including *Eubacterium rectale* and *Bifidobacterium* spp., predicted the response to anti-TNF-α medication in pediatric IBD patients (29). Thus, the gut microbiota may provide potential biomarkers for monitoring and predicting IBD treatment outcomes.

A global rise in IBD has been reported, especially in countries with previously low incidence rates, including China. To our knowledge, only a few studies have reported the characteristics of gut microbiota diversity in Chinese IBD patients (30, 31) and have mainly described correlations between shifts in microbial composition and disease phenotypes. Quantitative real-time PCR or denaturing gradient gel electrophoresis (DGGE), each targeting the 16S rRNA gene of selected bacteria, was used in those two Chinese studies. Due to the low throughput and low resolution of these methods, some key players of the microbial dysbiosis in IBD may not be discovered.

Using bar-coded 16S rRNA amplicon sequencing, we examined the gut microbiota of Chinese healthy individuals and patients with new-onset UC and CD before treatment initiation. We also included subjects representing a variety of phenotypes with respect to disease locations and activities. These data sets were compared with results from the RISK and PRISM IBD cohorts in the United States (9). This multicenter association study of gut microbiota and IBD, in which over 1,000 treatment-naive patients were included, represents the most comprehensive cross-cohort and cross-ethnic analysis involving this disease state performed to date. Additionally, we characterized the composition of fecal microbiotas from prospectively recruited patients with CD prior to and after receiving IFX treatment. The aims of this study were to identify gut microbiome patterns in Chinese IBD patients with different disease activities and statuses, to discover homogeneity and heterogeneity of IBD gut microbiota patterns in different populations, and to find out if there are any universal and specific biomarkers in gut microbes which can indicate and predict disease progression and IFX treatment responses.

## RESULTS

### Dysbiosis of gut microbiota patterns in Chinese IBD patients.

We recruited 72 CD patients, 51 UC patients, and 73 healthy volunteers who were members of the Han ethnic group living in China. The clinical characteristics of the participants are shown in Table S1 in the supplemental material. Sixteen patients with active CD received IFX treatment and were followed for up to 30 weeks posttreatment.

10.1128/mSystems.00188-17.8 TABLE S1  Baseline clinical characteristics of the subjects. Download TABLE S1, DOCX file, 0.1 MB. Copyright © 2018 Zhou et al. 2018 Zhou et al. This content is distributed under the terms of the Creative Commons Attribution 4.0 International license.

A total number of 1,376,142 high-quality 16S rRNA gene sequences were obtained for 196 samples from the cross-sectional study, with an average of 14,042 ± 7,304 (mean ± standard deviation [SD]) sequences per sample.

**FIG 1:**
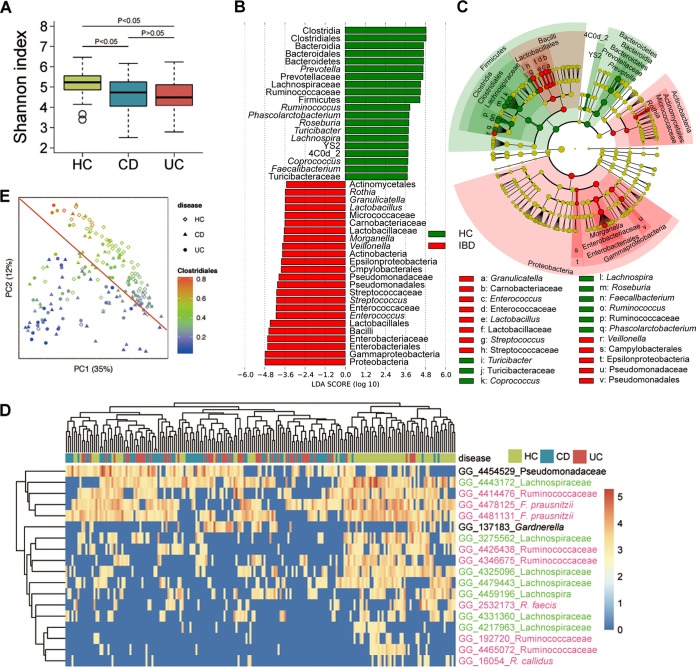
Dysbiosis of gut microbiota patterns in Chinese patients with IBD. (A) Comparison of Shannon index values between HC, CD, and UC groups. (B) Taxa listed according to their linear discriminant analysis (LDA) values determined from comparisons between the HC and IBD groups as computed by the use of the LEfSe algorithm. (C) Taxa illustrated according to their taxonomic relationship using a cladogram, showing the discriminative patterns in taxonomic lineages. The LDA cutoff values for panels A and B were set at 3.5. (D) Heat map showing abundance distributions for the 18 operational taxonomic units (OTUs) identified as key variables using the Kruskal-Wallis test (after Bonferroni correction) among the HC, CD, and UC groups. *Lachnospiraceae* OTUs are colored in green, and those of *Ruminococcaceae* are colored in red. (E) Weighted UniFrac PCoA data showing grouping patterns of HC, CD, and UC. Each dot represents a sample, with shapes indicating groups and colors the abundance of *Clostridiales*.

Consistent with previous reports (6, 32), the levels of alpha diversity of both CD and UC in our cohort were markedly reduced compared to the levels seen with healthy controls as indicated by the Shannon index (Fig. 1A). *Firmicutes*, *Bacteroidetes*, and *Proteobacteria* were the most abundant phyla, together accounting for up to 95% of the sequences on average, while *Actinobacteria*, *Fusobacteria*, *Verrucomicrobia*, *Tenericutes*, *Synergistetes*, and *Cyanobacteria* each accounted for 0.1% to 5% of sequences (see Fig. S1A in the supplemental material). Genus-level characterization is more complex, as the 20 most abundant genera observed in our study constituted only up to 60% of the total microbiome, with *Bacteroides* dominating the composition (Fig. S1B). At both the phylum and genus levels, we observed that the microbial composition seen with both CD and UC patients was different from that seen with the HC group (Fig. 1B and C; see also Fig. S2A and B). We used Kruskal-Wallis analysis combined with Bonferroni adjustment for multiple comparisons to screen the gut microbiome differences at the operational taxonomic unit (OTU) level between HC, CD, and UC. A total of 18 OTUs were significantly enriched in healthy controls and reduced in both CD and UC patients. These OTUs distinguished healthy controls from IBD patients, although they did not enable a distinction between patients with CD and UC (Fig. 1D and Fig. S2C), and the same groups were similarly indistinguishable in the Gevers RISK cohort (Fig. S2D). Most of these OTUs belonged to the order *Clostridiales*. This was also confirmed by the use of a linear discriminant analysis effect size (LEfSe) algorithm, showing that it was the most significantly enriched taxon in the healthy individuals (Fig. 1C and D). Specifically, levels of members of the family *Lachnospiraceae*, including the genera *Roseburia* and *Coprococcus*, were depleted under IBD conditions. This result accords with previous studies showing reduction of *Clostridiales* levels in IBD microbiota (33). Unsupervised clustering using principal-coordinate analysis (PCoA) based on weighted UniFrac distance data (34) also showed that the gut microbiotas of IBD differed significantly from those of healthy controls (HC) (analysis of similarity [ANOSIM] test, *P* = 0.001) and that the HC samples showed greater *Clostridiales* enrichment (Fig. 1E).

10.1128/mSystems.00188-17.1 FIG S1  Microbial composition at the phylum level (A) and genus level (B) of HC, CD, and UC groups. Download FIG S1, TIF file, 0.7 MB. Copyright © 2018 Zhou et al. 2018 Zhou et al. This content is distributed under the terms of the Creative Commons Attribution 4.0 International license.

10.1128/mSystems.00188-17.2 FIG S2  Discriminative taxa determined by LEfSe (the LDA cutoff value was set at 3.5) between HC versus CD groups (A) and HC versus UC groups (B). The supervised classification analysis was performed using random forest analysis to distinguish between the UC and CD groups in the present Chinese cohort (C) and in the RISK cohort of Gevers et al. (D). Download FIG S2, TIF file, 1.7 MB. Copyright © 2018 Zhou et al. 2018 Zhou et al. This content is distributed under the terms of the Creative Commons Attribution 4.0 International license.

### The alteration of gut microbiota in Chinese IBD patients is consistent with that of Westerners.

**FIG 2:**
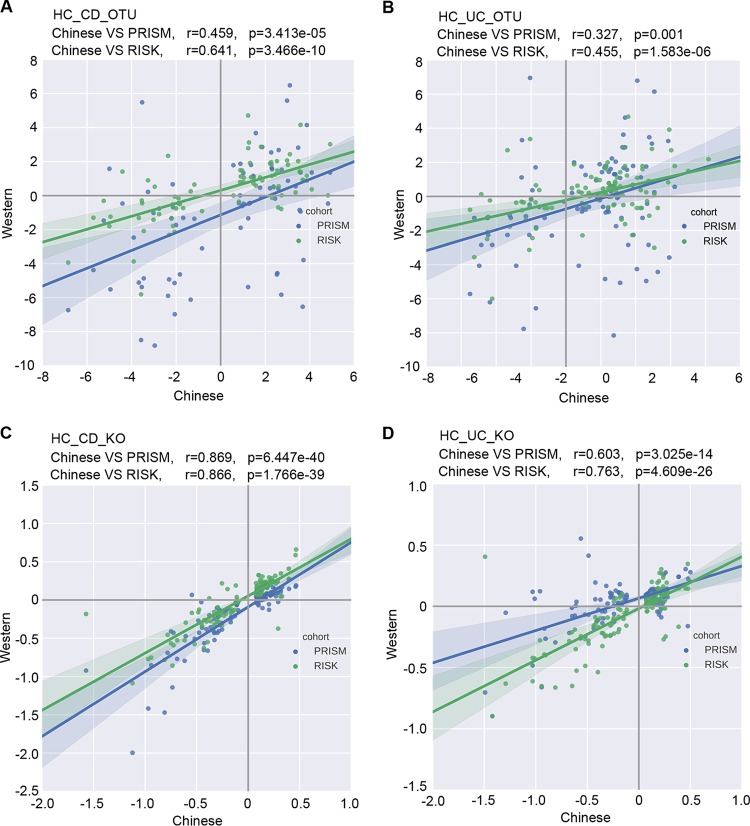
The similar shifts of gut microbiota in IBD across cohorts. Comparisons of OTUs (A and B) and predicted KOs (C and D) differentiated between healthy controls and subjects with IBD in the current study and the RISK and PRISM cohorts with biopsy samples. Each dot represents an OTU or KO that differed significantly between healthy and disease samples in Chinese cohorts. Axes indicates the log2-fold changes of the levels of these OTU/KO abundances between subjects with disease and healthy individuals in the Chinese cohort (*x* axis) and the Western cohorts (*y* axis), with the RISK cohort indicated in green and the PRISM cohort in blue. The correlation coefficients and the *P* values determined from comparisons between the cohorts are labeled on the plot.

It is well known that host lifestyle affects gut microbiota. The gut microbiotas harbored by the Chinese population are different from those harbored by the Western population (35). Additionally, samples from different studies of gut microbiota are generally clustered by study due to the technical variations in sample collecting and processing. Thus, it is not surprising that the Chinese samples were separated from the Western samples in the PCoA when we combined data from this study with data from the cohorts studied by Gevers et al. (9) (Fig. S3). Despite the overall microbial difference across the studies as shown in the PCoA plot, the results of the differential abundance analyses described above suggest that the microbial shift in Chinese IBD patients, compared to HC patients, may resemble that in Westerners. To examine this further, we selected OTUs that differed in relative abundances between HC and CD in our Chinese cohort, on the basis of a permutation test performed with a false-discovery rate (FDR) of less than 0.1 (the criterion was mildly relaxed to include more OTUs for this analysis). The log_2_-fold changes in these OTU abundances between the CD and HC groups were computed and plotted against those from biopsy samples of RISK and PRISM cohorts. As shown in Fig. 2A, these OTU abundance changes were highly correlated across these cohorts (Spearman correlation coefficient *r* = 0.459, *P* value = 3.41e−5 for PRISM; *r* = 0.641, *P* value = 3.47e−10 for RISK). A similar universal pattern was also seen in the UC cohorts (*r* = 0.327, *P* value = 0.001 for PRISM; *r* = 0.455, *P* value = 1.58e−6 for RISK) (Fig. 2B). However, stool samples from the two Western cohorts showed much less resemblance (Fig. S4A and B). The reasons for these differences are unclear; they could even have arisen from different practices in sample collection and processing. Samples were collected from the midstream stool in the present study, which is less convenient than swabbing but may retain the signal of microbial changes in IBD patients better, due to the biogeographic heterogeneity in the stool.

10.1128/mSystems.00188-17.3 FIG S3  PCoA based on unweighted UniFrac distance indicates that the Chinese samples (including HC, CD, and UC) are separated from the cohorts of Gevers et al. Download FIG S3, TIF file, 1.1 MB. Copyright © 2018 Zhou et al. 2018 Zhou et al. This content is distributed under the terms of the Creative Commons Attribution 4.0 International license.

10.1128/mSystems.00188-17.4 FIG S4  The similar shifts of gut microbiota in IBD across ethnicities. Comparison of OTUs (A and B) and predicted KOs (C and D) differentiated between the healthy and IBD subjects in the current study and the study by Gevers et al. performed with stool samples. Each dot represents an OTU or KO statistically significantly different between healthy and disease samples in Chinese cohorts. Axes indicates the log2-fold changes of these OTU/KO abundances between disease subjects and healthy individuals in the Chinese cohort (*x* axis) and Western cohorts (*y* axis), with RISK data indicated in green and PRISM data in blue. The correlation coefficients and the *P* values determined from comparisons between the cohorts are labeled on the plot. Download FIG S4, TIF file, 0.7 MB. Copyright © 2018 Zhou et al. 2018 Zhou et al. This content is distributed under the terms of the Creative Commons Attribution 4.0 International license.

Furthermore, we predicted KEGG orthology (KO) data from 16S rRNA amplicon taxonomic profiles using PICRUSt and found that the KO abundances changed similarly across cohorts, reflecting the patterns that we saw at the OTU level (Fig. 2C and D; see also Fig. S4C and D). These patterns were even more consistent in the KEGG orthologues than the OTUs, suggesting that there are some variations among cohorts and ethnic groups in OTU composition, while those OTUs seem to provide similar functions. Specifically, the pathways that increased in members of both the CD and UC groups included xenobiotic degradation (caprolactam degradation, limonene and pinene degradation, and toluene degradation), amino acid metabolism (tryptophan metabolism and lysine degradation), and electron transfer carriers; in contrast, the decreased pathways included microbial motility (bacterial chemotaxis, bacterial motility proteins, and flagellar assembly), germination, and sporulation.

**FIG 3:**
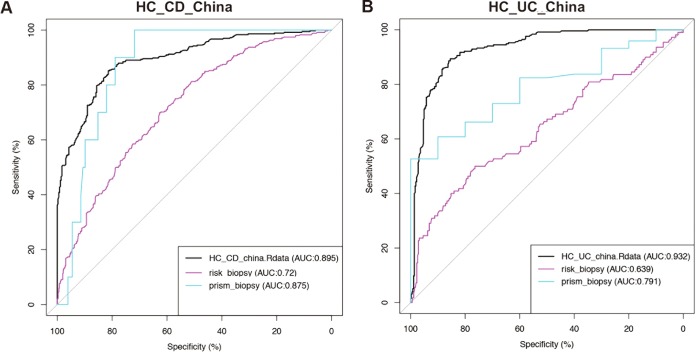
Gut microbiotas distinguish diseases from health similarly across cohorts. The black ROC curve indicates the accuracy of the classification model built on the Chinese cohort for classifying HC versus CD (A) and HC versus UC (B). Colored curves are the classification accuracies when these models are applied to the other cohorts.

To explore the possibility of using the observed OTU changes to identify IBD, we built supervised classification models based on Chinese samples and evaluated the accuracies of the models with 5 repeats of 10-fold cross-validation. The gut microbiota is informative enough to distinguish HC samples from CD and UC samples with model accuracy of 89.5% and 93.2%, respectively. Similarly, the model built from RISK and PRISM biopsy samples achieved high prediction accuracies as well, although Western fecal samples are less informative for classification of IBD from HC (Fig. S5), in concordance with the findings in the correlation analysis described above. Additionally, to investigate whether the model can be applied across cohorts, we tested a model trained by the use of Chinese samples on the RISK and PRISM samples. The prediction accuracy across cohorts was reduced only marginally. For example, the predictive model constructed using Chinese CD stool has an 87.5% accuracy level in predicting PRISM CD biopsy samples and the model trained using Chinese UC stool has a 79.1% accuracy level in predicting PRISM UC biopsy samples (Fig. 3). The RISK cohort is less well predicted than the PRISM cohort, likely because the RISK samples were mainly from children and adolescents instead of adults. Consistent with Fig. 2 and Fig. S4, the biopsy samples are better predicted with the Chinese model than the fecal samples (Fig. S6A and B). Taken together, these findings suggest that there are consistent changes in gut microbiota of IBD patients across populations and that they can serve as universal biomarkers for the classification of IBD states (32).

10.1128/mSystems.00188-17.5 FIG S5  Supervised classification of the HC group and the CD and UC groups using biopsy or stool samples from the RISK cohorts. The PRISM cohort does not have sufficient samples to build a robust classification model through cross-validation. Download FIG S5, TIF file, 0.5 MB. Copyright © 2018 Zhou et al. 2018 Zhou et al. This content is distributed under the terms of the Creative Commons Attribution 4.0 International license.

10.1128/mSystems.00188-17.6 FIG S6  Gut microbiotas distinguish IBD subjects from healthy subjects similarly across cohorts. The black ROC curve indicates the accuracy of the classification model built on data from the Chinese cohort for classifying the HC data versus the CD data (A) and the HC data versus the UC data (B). Colored curves represent the prediction accuracies seen in applying this model to the other cohorts. Download FIG S6, TIF file, 1.4 MB. Copyright © 2018 Zhou et al. 2018 Zhou et al. This content is distributed under the terms of the Creative Commons Attribution 4.0 International license.

### Gut microbiota signatures associated with disease activities.

**FIG 4:**
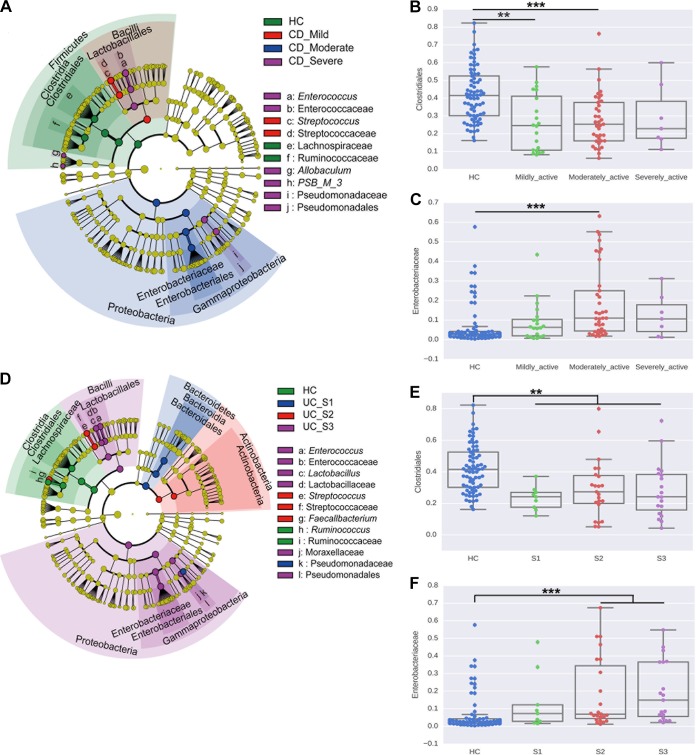
Bacterial biomarkers associated with disease severity. (A) Cladogram representing taxa with different abundances for CD activity. The size of each circle is proportionate to the abundance of the taxon. (B and C) Relative abundances of *Clostridiales* (B) and *Enterobacteriaceae* (C) in CD activity groups are shown. (D) Cladogram representing taxa with different abundances for UC activity. (E and F) Relative abundances of *Clostridiales* (E) and *Enterobacteriaceae* (F) in UC activity groups are shown. The statistical significance was determined with the Wilcoxon test and was adjusted using the false-discovery rate (FDR). **, *P* value < 0.01; ***, *P* value < 0.001.

We further analyzed the characteristics of gut microbiota in different disease activity subgroups of IBD patients. LEfSe results showed larger proportions of *Bacilli*, represented by *Streptococcus*, in patients with mild CD compared to other groups. Significant enrichment in *Proteobacteria* and *Enterococcaceae* (Fig. 4A and C) and depletion in *Ruminococcaceae* and *Clostridiales* (Fig. 4A and B) were seen in patients with moderate to severe CD. Levels of *Bacteroidetes*, represented by *Bacteroidia*, and *Pseudomonadaceae* were enriched in patients with mild UC (Montreal classification of severity of ulcerative colitis score, S1). *Streptococcus* levels were increased in patients with moderate UC (score, S2), resembling mild CD. Species of the *Proteobacteria* phylum and *Bacilli* class were enriched in patients with severe UC (score, S3) (Fig. 4D and F). *Clostridiales* levels were decreased in all active UC patients (Fig. 4E). Notably, a majority of these differences in microbiota with regard to disease activity were related to the *Firmicutes*, *Bacteroidetes*, and *Proteobacteria* phyla (Fig. 4A and D).

Crohn’s disease may lead to a stricture phenotype and penetrating complications, which indicate disease progression and impact the efficacies of treatments (27). In an advanced stage, CD can induce fistulas, i.e., abnormal passageways created between the bowel and other body parts. They often cause severe impairment in the patient’s quality of life (36). To determine whether any of the microbes were associated with these disease behaviors, we used the LEfSe algorithm for analysis and found that *Enterobacteriaceae* and *Pseudomonadaceae* were enriched in stricturing CD (CD_B2 [Montreal classification of stricturing behavior of Crohn’s disease]), while levels of *Aeromonadaceae* in the *Proteobacteria* phylum were enriched in penetrating CD (CD_B3 [Montreal classification of penetrating behavior of Crohn’s disease]) (Fig. S7A). *Enterococcaceae* and *Pseudomonadaceae* were the key taxa enriched in fistulizing CD patients (Fig. S7B). These results also show that the increase in *Proteobacteria* (*Enterobacteriaceae*) was strongly correlated with CD severities.

10.1128/mSystems.00188-17.7 FIG S7  Biomarkers determined for CD behavior (A) and for CD behavior complicated by the presence of fistulas (B) using LEfSe and illustrated by cladogram. The LDA cutoff value was set at 2. Download FIG S7, TIF file, 0.9 MB. Copyright © 2018 Zhou et al. 2018 Zhou et al. This content is distributed under the terms of the Creative Commons Attribution 4.0 International license.

### The gut microbiota is restored during disease remission, and certain microbes, especially *Clostridiales*, enabled predictions of the response of IFX treatment in CD.

**FIG 5:**
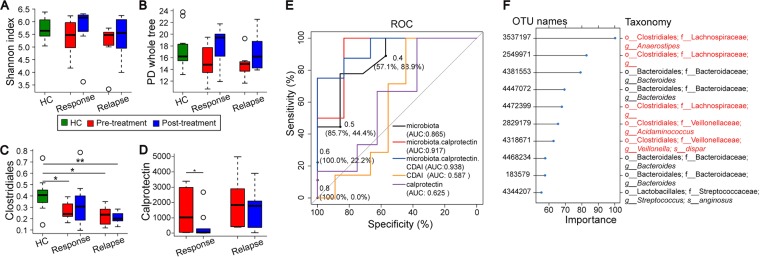
The microbiotas differ in the response and the relapse groups of CD patients treated with infliximab (IFX). The alpha diversity, measured by Shannon index (A) and PD whole tree (B), *Clostridiales* abundance (C), and calprotectin abundance (D), was restored in the response group after treatment compared to the relapse group. Stars (*) indicate *P* values of <0.05. (E and F) Supervised prognostic prediction of Crohn’s disease progression (IFX response or relapse). (E) The accuracy of prediction was best using the combination of microbiota, calprotectin, and CDAI (93.8%). The use of the microbiota alone (86.5%) still outperformed the results seen with the traditional clinic markers of CDAI (58.7%) or calprotectin (62.5%). (F) The top informative OTUs that contribute to the classification model. *Clostridiales* OTUs, colored in orange, are the most informative OTUs.

We followed 16 CD patients treated with IFX to week 30 to explore if the gut microbiotas were restored after IFX treatment and whether there were any microbial differences between IFX response and IFX relapse patients. A total number of 1,646,642 sequences were obtained from 27 fecal samples (including the 11 HC samples described above) for this longitudinal analysis, with an average of 15,106 ± 6,902 (mean ± SD) sequences. After initial IFX-induced remission, relapse occurred in 43.75% (7/16) of patients when reexamined at the end of week 30. The IFX treatment alleviated disease activity and increased microbial alpha diversity, measured by both Shannon index and PD whole tree (Fig. 5A and B), in the response group and, to a lesser extent, in the relapse group. The level of *Clostridiales*, the reduction of which was found as a signature of IBD (Fig. 1D and E; see also Fig. 4B and E), was not detected to be statistically significantly different from that of HC after IFX treatment in the response group, indicating its restoration after the IFX-induced response (Fig. 5C). These results imply that the *Clostridiales* reenrichment was correlated with disease remission after treatment and could potentially be used as a biomarker to guide treatment. The level of calprotectin, which has been recommended as a biomarker for IBD activity and prognosis, was also decreased more in the response group than in the relapse group after treatment (Fig. 5D).

To further test whether the gut microbiota provides biomarkers for prognosis of IFX treatment for CD patients, we derived and evaluated a model trained on the gut microbiota at baseline (at week 0) to predict the IFX-induced outcome (response or relapse) at week 30. The use of the microbiota alone improved the prediction to 86.5% accuracy, compared with that determined with the Crohn’s disease activity index (CDAI) (58.7%) and the level of calprotectin (62.5%), both of which are conventionally used to assess treatment effectiveness in clinic. The use of microbiota data in combination with calprotectin and CDAI data can further improve the accuracy of prediction of the prognosis (to 93.8%) (Fig. 5E). The most informative features that contribute to the prognosis model include multiple *Clostridiales* OTUs (Fig. 5F). These results highlight the advantage of using gut microbiota to stratify IBD patients and to apply personalized treatment for optimal outcomes, although the data warrant further verification in a larger cohort(s).

## DISCUSSION

Conventionally, IBD is regarded as a Western disease. However, following the path of Western countries, the IBD incidence in Asian populations has been increasing, and IBD has increasingly become a global health care problem over the past decade (37). Although the exact etiology of IBD remains elusive, it is widely accepted that various factors, including host genetic background, gut microbiome, and environmental triggers, contribute to the onset of IBD symptoms (2, 3). People who have certain variant alleles of genes (such as NOD2 and interleukin-23 receptor [IL23R]) are more prone than others to developing IBD (4, 5, 38). Epidemiology evidence has also shown that smoking, diet, appendectomies, and stress have a complicated impact on IBD (1). The distinct genetic backgrounds of emerging IBD populations without risk gene alleles emphasize the role that environmental factors play in IBD pathogenesis. There is no doubt that the human gut microbiome is a key player in this process as a consequence of interacting with the immune system (1). For example, *Bacteroides fragilis* can secrete capsular polysaccharide A to induce expression of interleukin-10 from regulatory T cells and protect mucous from colitis in a NOD2- and ATG16L1-dependent way (39).

In this study, we characterized dysbiosis in a Chinese IBD population. We found that gut microbial diversity was reduced in IBD patients compared with healthy controls, with a nonsignificant trend toward a greater reduction of diversity in UC patients than in CD patients. These findings are coherent with those of previous research on colonic mucosa-associated bacterial microbiota (32). Our results comparing the gut microbiota from different populations demonstrated that the microbial alteration patterns of both Chinese and Western IBD patients are consistent with each other, as shown by the cross-cohort and cross-ethnicity meta-analyses. To the best of our knowledge, this was the first attempt to compare microbiota communities in Chinese and Western IBD populations. This report conceptually proves the potential of the use of the gut microbiome in one cohort to help diagnose and evaluate IBD status in other cohorts. It will therefore be of great value in clinical trials across multiple populations in IBD management, a major unsolved challenge.

Infliximab has been proven to be more effective in the treatment of CD and UC than some treatments using traditional medicines such as corticosteroids and thiopurines in previous studies (40, 41), but some issues still need to be addressed, including which population benefits most, when the therapy should be stopped, and whether the therapy is still effective if clinical relapse occurs (27). There are many factors associated with disease relapse or response such as demographic variables (including smoking, old age, and long duration of steroids), clinical variables (including CDAI scores and longer duration of disease), laboratory variables (including CRP and calprotectin), and IFX-related variables (IFX doses, serum IFX concentration, and IFX antibodies). However, these factors are *post hoc* or retrospective. In this study, we followed up the CD patients who received scheduled infliximab and analyzed their fecal microbiota before and after treatment to explore the potential predictors for CD clinical relapse based on gut microbial composition. We found that imbalanced microbial diversity and reduced *Clostridiales* abundance in CD patients were restored in patients who responded to infliximab treatment. Moreover, the use of the gut microbiota, alone or together with calprotectin and CDAI data, enabled more-effective prediction of infliximab treatment outcomes, although more samples are needed to confirm and improve this model before it can serve in clinical practice. These findings may help establish a set of microbiota-based biomarkers for predicting treatment efficacy for IBD, which may pave the way to the usage of gut microbiota to stratify IBD patients and apply personalized therapy for optimal outcomes.

Interestingly, although species of *Clostridiales* are depleted in IBD patients, CD patients with a relatively higher abundance of *Clostridiales* respond better to IFX treatment than those with lower abundance. During remission, *Clostridiales* is restored to close to the abundance level of healthy individuals. This indirectly suggests the protective role of the taxa in IBD pathogenesis. Many commensal *Clostridiales* species are well-known defensive symbionts. They can suppress proinflammatory bacteria (42), produce short-chain fatty acids (SCFAs) (43), and induce an immune response (44). The suppression of these fermentation-related bacteria causes a decline in SCFA production, resulting in increased colonic pH and ammonia production and absorption in the intestine (45). For example, *Faecalibacterium prausnitzii* is a well-described anti-inflammatory organism that is considered to be a health-promoting bacterium (46). Reduced abundance of *Faecalibacterium prausnitzii* has been associated with a higher rate of IBD recurrence (46). However, it is still unknown what other strains protect against IBD in what capacity, which needs further mechanistic investigation.

In conclusion, our report reveals congruence in the gut microbiome dysbiosis in IBD patients in cross-cohort and cross-ethnicity groups. These findings may aid the establishment of principles guiding IBD treatment. Our results reinforce the idea that the gut microbiota contains promising biomarkers for the noninvasive evaluation of IBD activity and assessment of therapeutic responses. The identification of disease activity-associated microbiome is a step toward establishing a set of microbiota-based biomarkers for the assessment of treatment and progression of inflammatory bowel disease.

## MATERIALS AND METHODS

### Ethics statement.

The Ethics Committee of Nanfang Hospital, Southern Medical University, approved this study (NHMEC2013-081). Patients were included in the study after providing written consent.

### Patients and samples.

Patients with CD or UC who had not received any treatments for those conditions were recruited for this study between June 2012 and July 2013 in the Department of Gastroenterology of Nanfang Hospital, Southern Medical University, China. Healthy volunteers at age 20 to 40 (to match the age and gender of patients with CD) were recruited from the adjacent community. Exclusion criteria were receipt of IBD treatment, age <18 years, receipt of antibiotics or probiotics within the previous 4 weeks, other known chronic disease, and pregnancy or breastfeeding status. Sixteen patients with active CD who received treatment with IFX (Remicade; Cilag AG, Schaffhausen, Switzerland) (5 mg/kg of body weight) at weeks 0, 2, 6, 14, 22, and 30 were followed up for 30 weeks.

All enrolled patients underwent colonoscopies for diagnostic purposes. Fecal samples (from midstream stool; both the first-stream stool and the last-stream stool were discarded to toilet) were collected from all enrolled subjects at hospital and stored at −80°C before further processing.

### Diagnostics of IBD diseases.

Diagnoses of UC and CD were based on the internationally accepted Lennard-Jones criteria (47). According to the Montreal classification (48), UC was classified as ulcerative proctitis (E1), left-sided (distal) UC (E2), or extensive UC (pancolitis; E3), based on the extent of the disease. CD was classified as located in the ileum (L1), colon (L2), or ileocolon (L3). Disease behavior (B) for CD was also classified as B1 (nonstricturing, nonpenetrating), B2 (stricturing), and B3 (penetrating).

For the evaluation of disease activity, the Mayo score (41) for UC and the Crohn’s disease activity index (CDAI) score (49) for CD were determined to estimate UC and CD activity (mild [S1], moderate [S2], or severe [S3]).

### Evaluation of clinical outcome following infliximab treatment.

Patients receiving IFX treatment underwent endoscopy at baseline and after 30 weeks of treatment. For the evaluation of disease activity and response to IFX therapy, the CDAI was determined prior to each IFX infusion through the last follow-up visit (at week 30). CRP level, erythrocyte sedimentation rate, white blood cell count, and neutrophil ratio were also determined.

Clinical response was defined as a reduction of ≥70 points in the CDAI after infusion. Clinical remission was defined as a CDAI value of <150. Clinical relapse during follow-up was defined as worsening of symptoms and a CDAI value of >150, with an increase of ≥70 points compared with the CDAI value at remission; the need for an additional steroid or IFX course; or the need for surgical resection. All other outcomes were defined as nonresponse (50).

### Fecal calprotectin assay.

Fecal calprotectin concentrations were measured with a quantitative PhiCal enzyme-linked immunosorbent assay (ELISA) kit (Immundiagnostik AG, catalog no. K6927) according to the manufacturer’s instructions. Fecal specimens were diluted 1:2,500. ELISA plates were read by the use of a Thermo Scientific microplate reader (Multiskan FC; optical density at 450 nm against 620 nm). Samples containing ≥100 μg of calprotectin per 1 g of feces were considered calprotectin positive (51).

### Total bacterial genomic DNA extraction.

Bacterial DNA was extracted from the fecal samples using a Tiangen stool DNA kit (Tiangen Biotech, Beijing, China), according to the manufacturer’s instructions (52). DNA concentrations were determined using a NanoDrop 2000 BioAnalyser (Thermo Fisher Scientific, Inc., Waltham, MA), and the remaining samples were stored at −20°C before PCR was performed.

### PCR amplification and Illumina sequencing.

We used bar-coded primers V4-515F (5′-GTGCCAGCMGCCGCGGTAA-3′) and V4-806R (5′-GGACTACHVGGGTWTCTAAT-3′) to amplify the bacterial 16S rRNA V4 fragments. The PCR cycle conditions were as follows: initial denaturation at 94°C for 2 min; 30 cycles of 94°C for 30 s, 52°C for 30 s, and 72°C for 40 s; and final extension at 72°C for 5 min. Each 25-μl reaction mixture consisted of 12.5 μl TaKaRa Premix Taq (D331A, version 2.0; TaKaRa Biotech, Dalian, China), 2 μl template DNA, 1 μl 10 μM bar-coded primer V4-515F, 1 μl 10 μM primer V4-806R, and 8.5 μl double-distilled water (ddH_2_O).

PCR products were gel purified using a QIAquick gel extraction kit (catalog no. 28704; Qiagen, Hilden, Germany) and sequenced using the 250-bp paired-ended strategy on an Illumina MiSeq system at Beijing Genomic Institute (BGI, Shenzhen, China).

### Bioinformatics analysis.

The raw sequences were quality controlled using QIIME v1.9.1 (53) with default parameters. The closed-reference OTU clustering was done at 97% similarity level against the GreenGenes database (v13_8) (54). After the samples were rarefied to the same sequencing depth, alpha diversity, beta diversity, and differential OTU abundance analyses were performed with QIIME, PICRUSt (55), and LEfSe (56) tools.

To compare the IBD effects across ethnic groups, the sequences from RISK and PRISM cohorts of patients in the United States were downloaded from qiita.ucsd.edu (study identifier [ID]: 1939). All the sequences in these two studies were trimmed to the same length of 150 nucleotides (nt) on the same region of the 16S rRNA gene to minimize the technical variation. A single closed-reference OTU picking run was done on the combined sequences.

Random forest classification models were trained on features of the OTU data using the caret R package (57) with 5 repeats of 10-fold cross-validation, except for the IFX outcome classification, which was performed using leave-one-out cross-validation due to the small sample size. The model was evaluated with the area under the curve (AUC) derived from receiver operating characteristic (ROC) curve analysis. ROC analysis was used to compensate for the uneven distribution of the three sample types in this study (58). Importance scores of a model were determined for each feature based on the increase in prediction error when that feature was randomly permuted while all others were left unchanged (59).

The functional profile of KEGG orthology (KO) for each sample was predicted from 16S data with PICRUSt (55). The predicted KO abundances are collapsed to level 3 by grouping them into a higher level of functional categorization.

### Data availability.

Data were deposited in ENA under accession number PRJEB22028.
