# Supplementary material for: Gut Microbiota Offers Universal Biomarkers across Ethnicity in Inflammatory Bowel Disease Diagnosis and Infliximab Response Prediction
Source: mSystems. 2018 Jan 30;3(1):e00188-17. doi: 10.1128/mSystems.00188-17 (PMC5790872; doi:10.1128/mSystems.00188-17)
Supplement: TABLE S1 [file sys001182168st1.docx]

**Table S1** Baseline clinical characteristics of the subjects.

|  | **HC (n=73)** | | **CD (n=72)** | | **UC (n=51)** |
| --- | --- | --- | --- | --- | --- |
| Sex (female/male) | | 30/43 | | 33/39 | 23/28 |
| Age (years; mean±SD) | | 30.07±6.36 | | 31.81±12.668 | 41.75±14.362 |
| Duration (months; median) | | NA | | 24.00 | 29.5 |
| CDAI score (median) | | NA | | 258.62 | NA |
| Mayo score (median) | | NA | | NA | 8.00 |
| Disease activity [n (%)] | |  | |  |  |
| UC Mild (S1) | | NA | | NA | 8 (15.69%) |
| Moderate (S2) | | NA | | NA | 24 (47.06%) |
| Severe (S3) | | NA | | NA | 19 (37.25%) |
| CD Remission | | NA | | 7 (9.72%) | NA |
| Mild | | NA | | 21 (29.17%) | NA |
| Moderate | | NA | | 37 (51.39%) | NA |
| Severe | | NA | | 7 (9.72%) | NA |
| Extent of disease [n (%)] | |  | |  |  |
| UC Proctitis (E1) | | NA | | NA | 10 (19.61%) |
| Left-sided (E2) | | NA | | NA | 14 (27.45%) |
| Pancolitis (E3) | | NA | | NA | 27 (52.94%) |
| CD Ileum (L1) | | NA | | 24 (33.33%) | NA |
| Colon (L2) | | NA | | 14 (19.44%) | NA |
| Ileocolon (L3) | | NA | | 34 (47.23%) | NA |
| CD Behavior [n (%)] | |  | |  |  |
| non-stricturing,non-penetrating (B1) | | NA | | 33(45.83%) | NA |
| stricturing (B2) | | NA | | 25(34.72%) | NA |
| Penetrating (B3) | | NA | | 14(19.46%) | NA |
| Fistulising CD [n (%)] | | NA | | 12 (16.67%) | NA |

HC, healthy controls; CD, Crohn’s disease; UC, ulcerative colitis; SD, standard deviation; NA, not available/applicable; CDAI, Crohn’s Disease Activity Index.
