# Supplementary figures and images for: Gut Microbiota Offers Universal Biomarkers across Ethnicity in Inflammatory Bowel Disease Diagnosis and Infliximab Response Prediction
Source: mSystems. 2018 Jan 30;3(1):e00188-17. doi: 10.1128/mSystems.00188-17 (PMC5790872; doi:10.1128/mSystems.00188-17)

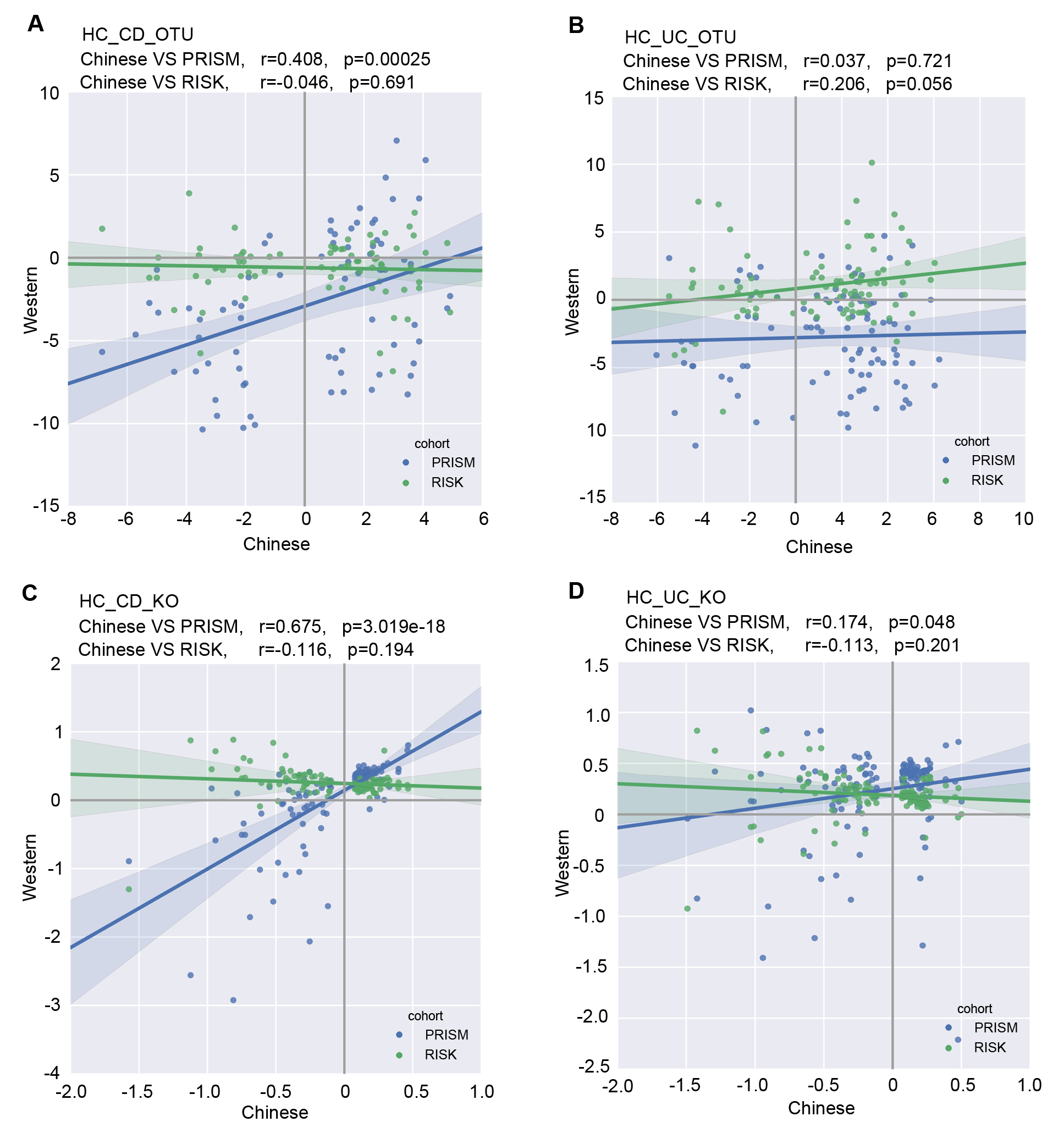

Supplement: FIG S4 [file sys001182168sf4.tif]

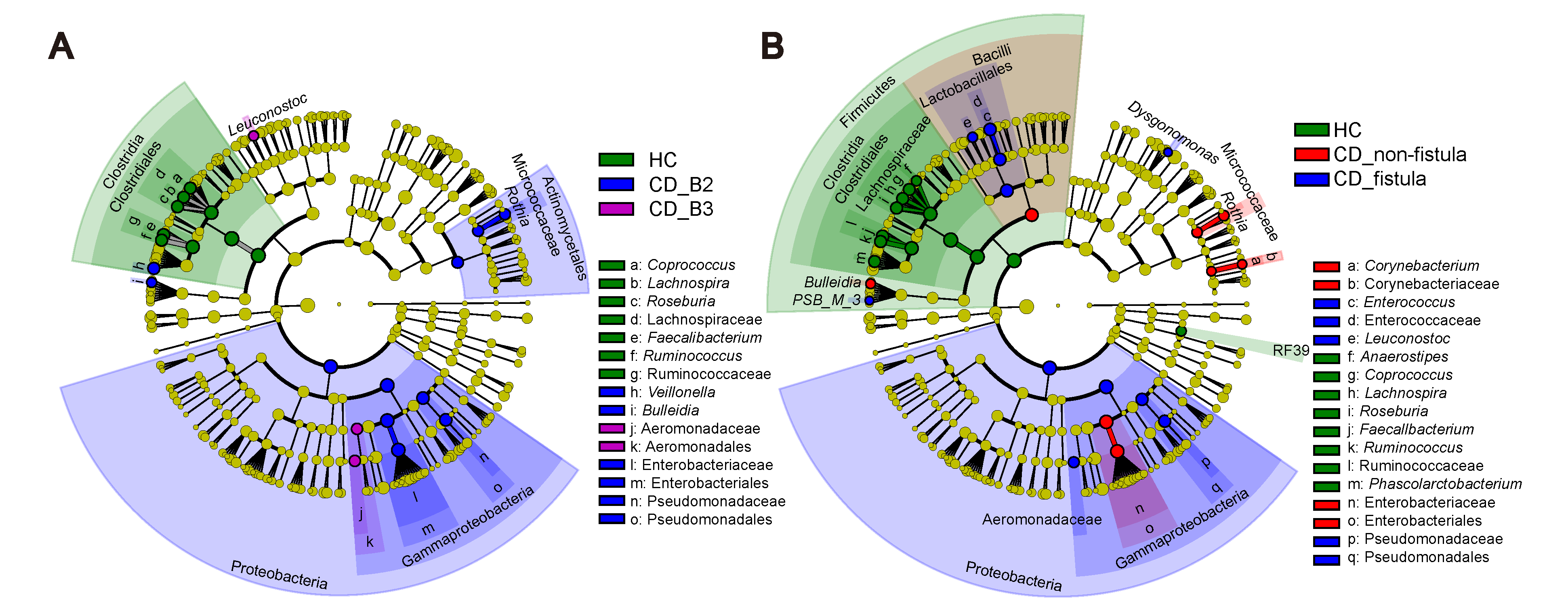

Supplement: FIG S7 [file sys001182168sf7.tif]
